# Dataset evaluating the effectiveness of the Konga model to address factors contributing to a low viral load suppression among children with HIV in Tanzania

**DOI:** 10.1016/j.dib.2023.109655

**Published:** 2023-10-10

**Authors:** Kihulya Mageda, Khamis Kulemba, Leornard K. Katalambula, Ntuli Kapologwe, Pammla Petrucka

**Affiliations:** a School of Nursing and Public Health, University of Dodoma, Dodoma, Tanzania; b Simiyu Regional Commissioners Office, Bariadi, Tanzania; c University of Saskatchewan, Saskatoon, Saskatchewan, Canada

**Keywords:** Viral load suppression, Antiretroviral therapy, HIV-positive children, Community-based intervention

## Abstract

Data were collected for a cluster-randomized clinical trial of the Konga community-based intervention using a validated questionnaire for children and caregivers. The raw and analyzed data include 82 participants with the following information: sociodemographic characteristics (caregiver's age, sex, and level of education, income, and caregiver's marital status) and clinical characteristics of the children (weight, CD4 cell count, and viral load at baseline and after 6 months of follow-up. The other data included in this dataset were weight, medication adherence, and opportunistic infections. Analysis of covariance (ANCOVA) was performed using the baseline VL. The outcome was viral load at the end of the intervention. Additionally, Omega squared (ω^2^) was used to calculate the effect size as an estimation of the strength of the intervention. These data will help researchers analyze data from similar studies and evaluate the effectiveness of community-based interventions for viral load suppression.

Specifications Table

**Table:** 

Subject	Infectious disease
Specific subject area	Care of human immunodeficiency virus (HIV) infection in children and treatment with antiretroviral therapy (ART)
Data format	Raw, Analyzed
Type of data	Table
Data collection	Data were collected using a questionnaire. The participants were children aged 2‒14 years with a viral load >1000 cells/mL and their caregivers. The sample size was 82 child–caregiver pairs, of which 41 were allocated to the intervention group and 41 to the control group.
Data source location	Institution: Simiyu Region Health System City/Town/Region: Simiyu-Bariadi Country: Tanzania Latitude and longitude: Latitude 2°1′ to 4° south of the equator, and longitude 33°3′ to 35°1′ east of the Prime (Greenwich) Meridian
Data accessibility	Repository name: Figshare Data identification number: https://doi.org/10.6084/m9.figshare.22141400.v2 Direct URL to data: https://figshare.com/articles/dataset/Untitled_ItemDataset_of_the_Konga_community-based_cluster-randomized_trial_in_Tanzanian_children_with_high_HIV_viral_loads/22141400
Related research article	M. Kihulya, L.K. Katalambula, N.A. Kapologwe, P. Petrucka, Effectiveness of a community-based intervention (Konga model) to address the factors contributing to viral load suppression among children living with HIV in Tanzania: a cluster-randomized clinical trial protocol, Biol Methods Protoc. 7 (2022) bpac002. https://doi.org/10.1093/biomethods/bpac002[1]

## 1 Value of the Data

• These data are useful because viral load (VL) measurement is essential for monitoring the effectiveness of antiretroviral therapy (ART) and is considered a surrogate marker for disease suppression. VL suppression (VLS) after early ART initiation is the primary treatment goal in children. This cluster-randomized clinical trial of children with a high VL (>1000 copies/mL) tested mitigating measures to improve VL suppression.
• These data will benefit researchers studying community participation in the care and treatment of children with HIV infection receiving ART. These data were used to test the effectiveness of the Konga model in improving VLS among children living with HIV and receiving ART. Despite their potential, community resources are often not engaged in providing support, and their roles remain undefined. Hence, these data will encourage service providers to engage with the community to promote retention and adherence to ART among children living with HIV.
• These data will be valuable to researchers comparing similar studies regarding community-based services as a contribution to reducing the HIV burden and mitigating its impact.
• The data will be valuable for training researchers in the analysis of covariance for analyzing experimental and observational data. Analysis of covariance is underutilized in health research.
• The data provide a framework for future in-depth studies on using community-based organizations (e.g., Konga) to overcome the challenges in HIV care, treatment, and prevention.
• The data provide a resource for leaders and policymakers to formulate effective policies for implementing HIV care and services.

## 2 Objective

Despite substantial antiretroviral therapy (ART) coverage in people living with HIV in Tanzania, viral load suppression (VLS) (viral load [VL] <1000 copies/mL) among HIV-positive children receiving ART remains unacceptably low. Of children enrolled in care and treatment centers (CTCs) and receiving ART, 82 % do not achieve VLS [2]. Through the National HIV/AIDS Control Program, the Government of Tanzania has tried to improve retention in care and ART adherence among people with HIV. These efforts have not been effective for VLS among children living with HIV receiving ART [3].

Therefore, we identified the need for a sustainable intervention promoting retention in care and adherence to ART and addressing the lack of VLS among children living with HIV in Tanzania. Thus, the main goal of this study was to identify a sustainable intervention that promotes retention in care and adherence to ART in children living with HIV receiving care from care and treatment centers (CTCs) in mainland Tanzania.

The objective of collecting these data was to evaluate the effectiveness of a community-based intervention (the Konga model) for addressing the factors contributing to low VLS among children living with HIV in Tanzania.

## 3 Data Description

HIV depletes CD4 cells, making individuals with HIV susceptible to illnesses that a healthy immune system would otherwise prevent [4], [5], [6]. The data presented in this article were collected from children with high VLs >6 months after ART initiation. The raw data are a set of demographic and clinical characteristics of children and are shared publicly in Figshare [7]. The data descriptor (Table 1) is provided with the shared dataset. The name of the dataset in the Figshare is the Konga Trial Database. It includes sociodemographic characteristics of children and their caregivers, health service and clinical characteristics before and after the intervention. The data include age, VL at the start and end of the study, distance from the child's home to the health facility, sex, education level, source of income, and ART adherence at the start and end of the study (Table 2). The adherence of children was measured using the pill count method, a standard method for measuring adherence in the study setting, and was performed by the ART nurse during the clinical visit. Adherence was recorded as “good adherence” if the child did not miss pills for more than 1 day and as “poor adherence” if the child missed pills for ≥2 days.

**Table 1:** Attributes and description of socio-demographic and clinical data obtained during the trial *(N=*82).

Attribute	Description
patient	Participant identification number
facility	The cluster in which the child was enrolled.
council	The district in which the child lives
konga	Indicator of assignment to the Konga intervention (treat) or control (con) cluster
age	Age of the child in years
pre_adhere	Adherence status of the child before enrolling in the study. (Adherence was measured using the pill count and was recorded in the database as “good adherence” if the child did not miss pills for more than 1 day and as “poor adherence” if the child missed pills for 2 or more days.
pre_VLR	Viral load before the start of the study
logpre_vlr	Logarithm Viral load before the start of the study
pre_weight	Weight before the start of the study
lastvrlwithresultstestdate	Date of last viral load measurement before the start of the study
begdate	The date on which the study started
dateconfirmedhivpositive	Date of diagnosis of HIV infection
post_VLR	Viral load at the end of the study
logpost_vlr	Logarithm viral load at the end of the study
post_adhere	Adherence status at the end of the study. (Adherence was measured using the pill count and recorded in the database as “good adherence” if the child did not miss pills for more than 1 day, and as “poor adherence” if the child missed pills for 2 or more days.
hvlresults	HIV viral load results
category	The stability category of the child at the start of the study. Patients were classified as stable or unstable: A stable client is a client who has been on ART for >6 months, age above 5 years, with a viral load <50 copies/mL, with good adherence, no history of opportunistic infection within the past 6 months, and not on a 3rd-line ART regimen.
prescription	Prescription given to the child at last visit
post_weight	Weight at the end of the study
censored	End of observation
educationlevelcaregivernum	Educational level of the caregiver
sex	Sex of the child
type	Relationship of the child to the caregiver
income	Source of income of the caregiver
tb_infection	Tuberculosis in the child detected after screening
enddate	Date on which the study ended

**Table 2:** Sociodemographic characteristics of children and caregivers before and after the intervention, N = 82.

Variable	Control	Intervention	Total	P-value
Age of the child (years), median [IQR]	8.9 [5.4–11.1]	8.7 [6–12]	8.8 [5.5–11.2]	
Pre-viral load (copies/mL), median [IQR]	12195 [6170–29300]	17400 [3600–73771]	13150 [3600–59200]	
Post-viral load (copies/mL), median [IQR]	65 [43–200]	20 [11–64]	50 [20–125]	
**Distance from home to the facility, *n* (%)**				
Living near (<10 km)	29 (78)	27 (67.5)	56 (73)	0.643
Living far (>10 km)	8 (22)	13 (32.5)	21 (27)	
**Sex, *n* (%)**				
Girl	19 (46)	19 (46)	38 (46)	>0.999*
Boy	22 (54)	22 (54)	44 (54)	
**Caregiver, *n* (%)**				
Both parents	4 (10)	11 (27)	15 (18)	0.242*
Father only	8 (20)	6 (15)	14 (17)	
Grandparent	5 (12)	7 (17)	12 (15)	
Mother only	19 (46)	12 (29)	31 (38)	
Other relative	5 (12)	5 (12)	10 (12)	
**Educational level (caregivers), *n* (%)**				
None	17 (41)	14 (35)	31 (38)	0.821*
Primary	23 (56)	25 (62.5)	48 (59	
Secondary	1 (2)	1 (2.5)	2 (2)	
**Source of income caregiver), *n* (%)**				
Small business	3 (7)	1 (2)	4 (5)	0.616*
Employed	0	1 (2)	1 (1)	
Peasant	38 (93)	39 (95)	77 (94)	
**Opportunistic infection (tuberculosis), *n* (%)**				
No	41 (100)	40 (98)	81 (99)	0.500*
Yes	0 (0)	1 (2)	1 (1)	
**Pre-adherence status, *n* (%)**				
Good	0 (0)	1 (2)	1 (1)	0.500*
Poor	41 (100)	40 (98)	81 (99)	
**Post-adherence status, *n* (%)**				
Good	31 (76)	40 (98)	71 (87)	0.007*
Poor	10 (24)	1 (2)	11 (13)	

⁎ Pearson's Chi-square test. IQR, interquartile range.

The data analysis was descriptive and inferential (Table 2, Table 3, Table 4).

**Table 3:** Fitted model of the viral load suppression results at the end of the study.

Sources	Partial SS	Df	MS	F	Prob > F
Model	5.8892732	2	2.9446366	6.61	0.0022
Konga	3.2534631	1	3.2534631	7.30	0.0084
Pre-viral load	2.9547811	1	2.9547811	6.63	0.0119
Residual	35.198618	79	0.44555213		
Total	41.087891	81	0.50725792		

Df, degrees of freedom; MS, mean square; SS, sum of squares.

**Table 4:** Measure of the effect size of the Konga intervention on viral load suppression.

Source	ω^2^	Df	90 % Confidence interval	
Model	0.1216458	2	0	0.2550703
Konga	0.0730236	1	0	0.2042359
Pre-viral load	0.0657669	1	0	0.1947727

Df, degrees of freedom.

## 4 Experimental Design, Materials and Methods

The National Council of People Living with HIV (NACOPHA) is a nonprofit, nongovernmental, national grassroots-based organization of people living with HIV (PLHIV) in mainland Tanzania. Since its establishment, NACOPHA has embarked on coordinating the efforts of PLHIV through their district clusters known as Konga to address the needs of PLHIV. Hence, in this study, we used a community-based model called the Konga model to provide services to children. The study was conducted in the Simiyu Region. It was a cluster-randomized clinical trial with concurrent intervention and control groups. The clusters comprised health facilities that provided HIV care and treatment. The randomization unit was the HIV care and treatment facility, and the unit of analysis was the patient. Cluster randomization was chosen for practical reasons and to prevent contamination according to patient or nurse preferences. Health facilities with a care and treatment center (CTC) were classified into three levels: hospitals, health centers, and dispensaries. Health facilities at each level were randomly assigned to the intervention or control group using computer-generated random numbers. The trial was conducted in 45 CTC clinics that delivered HIV care and treatment within the four selected districts (15 CTCs provided the intervention, and 35 CTCs provided standard care). All children living with HIV receiving ART at the 45 study CTCs with an HIV viral load (VL) >1000 cells/mL were recruited. We used the analysis of covariance (ANCOVA) method (Dekkers, 2016) to calculate the required sample size. We assumed that the correlation between the VL results at the start and end of the study would be 0.6, the mean of the intervention group 0, the mean of the control group 0.5, the power 80 %, and the standard deviation of both groups 1. Using the Stata function (sampsi 0.5, power (0.8) st1(1) std2(1) pre (1) r01(0.6)) method (ANCOVA)), the estimated sample size requirement was 82 (41 in the control group and 41 in the intervention group). Healthcare workers (i.e., ART nurses) identified and recruited children aged 2–14 years with a VL >1000 copies/mL. All children who met these conditions and were receiving ART from one of the study CTCs were eligible to be recruited. If the child was in an intervention cluster, personnel from the Konga visited the child's household to provide adherence counseling, psychosocial support, and screening for opportunistic infection. Standard operating procedures were developed and used to provide the Konga intervention. (The standard operating procedure for the Konga intervention is provided as a supplementary file.)

The study data were collected using a structured validated questionnaire for children and caregivers at baseline and after 6 months of follow-up. The inclusion criterion was children aged 2–14 years with a viral load > 1000 cells/mL attending a clinic. Stata software was used to analyze the data. An analysis of covariance (ANCOVA) was determined using the mean change in VL over the 6-month study period. The Omega-squared (ω^2^) value was used to calculate the effect size (an estimate of the strength of the treatment effect). F-tests and their corresponding p-values were used as measures of improvement.

## 5 Limitations

None.

## Ethics Statement

The protocol was reviewed and approved by the Institutional Ethics Clearance Board of the University of Dodoma on October 18, 2021 (reference number MA:84/261/02/A). Informed consent was obtained from the carers of all children in the study; the study was carried out in accordance with the Declaration of Helsinki.

## CRediT authorship contribution statement

**Kihulya Mageda:** Conceptualization, Methodology, Writing – original draft. **Khamis Kulemba:** Data curation. **Leornard K. Katalambula:** Conceptualization, Methodology, Supervision. **Ntuli Kapologwe:** Supervision. **Pammla Petrucka:** Writing – review & editing.

## Data Availability

Data set of the Konga Community-Based Randomized trialin Tanzanian Children with high HIV viral load (Original data) (Figshare)

## Appendix Supplementary materials

Image, application 1
