# Supplementary material for: Dataset evaluating the effectiveness of the Konga model to address factors contributing to a low viral load suppression among children with HIV in Tanzania
Source: Data Brief. 2023 Oct 10;51:109655. doi: 10.1016/j.dib.2023.109655 (PMC10590862; doi:10.1016/j.dib.2023.109655)
Supplement: Supplementary file 1 [file mmc1.pdf]

BIOLOGY Methods & Protocols

Biology Methods and Protocols, 2022, 1–6

https://doi.org/10.1093/biomethods/bpac002
Advance Access Publication Date: 7 January 2022
Protocols

PROTOCOLS

Effectiveness of a community-based intervention (Konga model) to address the factors contributing to viral load suppression among children living with HIV in Tanzania: a cluster-randomized clinical trial protocol

Mageda Kihulya [1,2,*], Leornard K. Katalambula[1], Ntuli A. Kapologwe [1,2] and Pammla Petrucka[3]

[1]School of Nursing and Public Health, University of Dodoma, PO Box 395, Dodoma 41210, Tanzania, [2]President's Office—Regional Administration and Local Government, PO Box 1923, Dodoma 41207, Tanzania and [3]Research & Graduate Studies, College of Nursing, University of Saskatchewan, 4400-4th Avenue, Regina, SK S4T 0H8, Saskatoon, Canada

*Correspondence address. School of Nursing and Public Health, University of Dodoma, PO Box 395, Dodoma, Tanzania. Tel: +255-735909001; E-mail: mageda121@gmail.com

Downloaded from https://academic.oup.com/biomethods/article/7/1/bpac002/6500270 by guest on 23 August 2023

Abstract

This study aims to test the effectiveness of a community-based intervention (Konga model) to improve viral-load suppression in children living with human immunodeficiency virus (HIV) and enrolled in care and treatment centers in Tanzania mainland. The study will be a cluster-randomized clinical trial study designed with both intervention and control arms. The study will involve 268 children with a viral load of >1000 copies/ml who are aged between 2 and 14 years. The children will be randomly allocated into the intervention and control arms. The intervention will include three distinct activities: adherence and retention counseling, psychosocial support, and comorbidity screening (i.e. tuberculosis). The outcome of the study will be assessment of the success of the intervention to increase medication adherence with the immediate result of reducing the viral load below 1000 copies/ml. Descriptive statistics will be used to calculate the mean, median, standard deviation, and interquartile range of continuous data. We will use frequencies and percentages to summarize categorical data. As for the primary outcome (proportion of HIV-infected children with viral suppression), we will compare the proportion of successful participants in the intervention and control arms. Proportions and tests for different proportions will be used as a measure of improvement. All statistical tests will be two-sided and $P < 0.05$ will be considered statistically significant.

Keywords: viral-load suppression; antiretroviral therapy; HIV-infected children; community-based intervention; Tanzania

Introduction

The retrovirus human immunodeficiency virus (HIV) attacks the body's cellular immune system and acquired immunodeficiency syndrome (AIDS) can eventually result [1]. The attack depletes CD4 cells, which leaves people vulnerable to illnesses that a healthy immune system would otherwise eliminate [2–4]. Retroviral infection remains the leading cause of morbidity and mortality throughout the world [5]. However, with the

Received: 18 November 2021; **Revised:** 22 December 2021; **Editorial Decision:** 27 December 2021; **Accepted:** 6 January 2022

© The Author(s) 2022. Published by Oxford University Press.
This is an Open Access article distributed under the terms of the Creative Commons Attribution License (https://creativecommons.org/licenses/by/4.0/), which permits unrestricted reuse, distribution, and reproduction in any medium, provided the original work is properly cited.

introduction to antiretroviral therapy (ART) and wide accessibility, mortality has been significantly reduced [6–8]. Thus, HIV infection has become a manageable chronic health condition, enabling people living with HIV (PLHIV) to live long and quality healthy lives [9].

Viral-load measurement emerged as an essential monitor of a therapy's effectiveness after ART initiation and it is considered a surrogate marker for disease progression [3, 10]. ART in children aims to suppress HIV replication and halt disease progression while reducing opportunistic infections and morbidities [11, 12].

Viral-load suppression (VLS) after early ART initiation is the primary goal in children [13]. Globally, about 400,000 children living with HIV under 15 years of age who are receiving ART and living in low- and middle-income countries have not achieved VLS [14]. In East-African nations, compared with other countries in sub-Saharan Africa, a low proportion of children on ART achieved VLS [15, 16]. Thus, there is high risk of developing AIDS. In Tanzania, program data and the Tanzania HIV Impact Survey (THIS) have demonstrated that VLS in pediatric patients continues to be low [4, 17, 18].

Several studies have cited factors causing unsuppressed viral loads, such as poor adherence and co-morbidities [14, 19, 20], malnutrition [21, 22], and underlying tuberculosis (TB) infection [23]. These patients who initially received ART still have advanced disease [24].

Despite substantial ART coverage in other groups living with HIV in Tanzania, the VLS among HIV-positive children on ART remains unacceptably low at 18%. That means that 82% of those enrolled in care and treatment centers (CTCs) and receiving ART did not achieve VLS [4]. Several studies have demonstrated that early enrollment of children to ART and maintaining good adherence (retention) reduces HIV replication and suppresses the virus [25, 26]. Through the National HIV/AIDS Control Program, the Government of Tanzania has made efforts to improve retention and adherence for PLHIV. These efforts have not however been effective for VLS among children due to the loss of follow-up of caregivers and their HIV-exposed infants [27].

Trials in different countries have demonstrated that community-based intervention improves HIV care services among children living with HIV and AIDS [25, 28–30]. Thus, UNAIDS [31] recommends a requirement of sustained engagement and unique inputs from various communities, from small informal groups at the grassroots level up to global coalitions.

We therefore identified the need for a sustainable intervention that promotes retention and adherence to ART and that addresses low VLS among children living with HIV in Tanzania.

Material and methods

Study area

We will conduct this study in the Simiyu Region. Administratively, the region is comprised of six district councils with a total of 218 health facilities (8 hospitals, 17 health centers, and 193 dispensaries), of which 106 sites provide ART.

Study design and population

We will use a cluster-randomized trial study designed with both intervention and control arms. The study will involve 268 children (134 controls and 134 patients who receive intervention) aged 2–14 years who are attending CTC and who have a viral load of >1000 copies/ml.

Recruitment

The healthcare workers (i.e. ART nurses) will identify and recruit children with a viral load of >1000 copies/ml and who were aged 2–14 years. Before recruitment, informed consent will be obtained from their caregivers.

Intervention

The UNAIDS and previous studies have recommended the engagement of communities, from small informal groups at the grassroots level up to global coalitions, to improve HIV care [31]. The National Council of PLHIV (NACOPHA) is a non-profit, non-government organization. It is a national grassroots-based organization of all individuals who are recognized through organized groups and clusters of PLHIV in Tanzania mainland. Since its establishment has embarked upon coordinating the efforts of PLHIV through their district clusters known as "Konga" to address the needs of PLHIV. Konga is a Swahili name meaning that is a Cluster of PLHIV at the district level, thus Konga is the smallest unit of NACOPHA. Hence, we will use the community of PLHIV known as Konga to provide services to children.

Plan for the intervention

Role of the health worker

The health workers, usually the ART nurses at the selected facility, will identify children with low VLS and link them to the Konga. They will also continue routine care of the child.

Roles of the Konga

We will provide the standard operating procedures (SOPs) to the Kong such that different person will use for the provision of the desired intervention. The SOP will have specific standards adapted from the National HIV care and treatment guidelines, thus the Konga will use the SOP:

- To provide enhanced adherence counseling and intensive follow-up. Here, the Konga will routinely visit the client's house and at every visit they will assess and document:
  - Medication regimen, storage of drugs at home.
  - Doses missed: how often, why, then solve problem with the client.
  - Side effects such as headache, nausea and vomiting, diarrhea, fatigue, sleeping, difficulty, a dry mouth, a rash, dizziness and a pain and client response.
  - Use of other medications, including traditional medicines.
  - Challenges.
- Follow-up screening for TB and other comorbidities (members of the Konga will visit the children and screen for opportunistic infection and encourage them to visit the health facility for further management); and
- Provision of psychosocial support by members of the Konga team.

Outcome measures

The study will have two outcome measures. The primary measure will be the adherence, this will be measured by using the

Downloaded from https://academic.oup.com/biomethods/article/7/1/bpac002/6500270 by guest on 23 August 2023

standard adherence tool (National AIDS Control Program [32]) and loss to follow-up (retention in CTC), which will be compared in both arms. We will measure these metrics at baseline, subsequent follow-up, and the study's end (i.e. 6 months). The secondary measure will be the viral load, which will be measured at the baseline and subsequently at the 6-month study's end, comparing both arms.

Sample size calculation and selection

The trial will be conducted in 20 CTC clinics that deliver HIV care and treatment within the four selected districts (10 CTCs will carry out the intervention and 10 CTCs will carry out standard care). We determined the study size from the primary outcome of proportional HIV-positive children with a viral load >1000 copies/ml. According to THIS (2018), 18% of HIV-positive children under the age of 15 years had a viral load >1000 copies/ml; thus, we assumed that value for standard care. Therefore, under a cluster design, the study will need a total sample size of about 268 children (134 in the intervention group and 134 in the control group) to increase the proportion of children with viral suppression from 18% to 30% with 80% power, ata5% significance level, an intra-cluster correlation of 0.01, and 10% expected loss to follow-up. We carried out this power calculation using the Stata™ function “clustersampsi” command (clustersampsi, binomial p1[0.18] p2[0.3]k[20] rho[0.01] alpha[0.05] beta[0.8]).

Selection and randomization of CTC

A site assessment will be performed before the final site selection and sites will be selected to participate in the study based on infrastructure feasibility, last numbers of children enrolled into CTC during the past year, numbers and qualification of staff affiliated to CTC and balanced representation of the different level of health facilities and urban versus rural affiliations. Assignment of sites to one of the two study arms will be done by randomization (Fig. 1). Stratification during the randomization process (e.g. rural versus urban) will be considered.

The study will be open-label whereby the Konga, caretakers, and the children will be aware of the intervention received since neither the outcome assessor nor person receiving the intervention will influence the outcome. The study's primary outcome is to reduce the viral load <1000 copies/ml.

Data collection

Baseline data

At the beginning of the study, we will collect baseline data using a checklist. We will collect information from caregivers and their HIV-affected children. From the caregiver, we will collect data, such as social demographic characteristics, age and sex, level of education, income, and marital status. We will collect data, such as age, sex, and clinical characteristics of the children, and also weight, CD4 count, and VLS. Other data collected from the child will include anthropometric measurements, nutrition status, medication adherence, and opportunistic infection status.

Follow-up data

We will follow participants monthly in both the intervention and control arms and observe adherence, retention, and opportunistic infections.

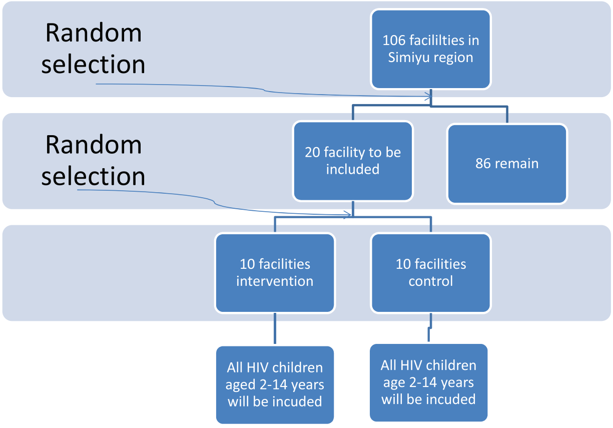

Figure 1: study design and flow chart.

End of study (6 months)

At the 6-month point, we will collect data from both arms regarding the viral load, weight, and number of missing doses (adherence).

Measurement of variables

Dependent variable

The dependent variable will be the viral load cell count and it will be classified as suppression when the count is <1000 copies/ml and unsuppressed when >1000 copies/ml.

Independent variable

The independent variables will be age, weight, adherence, and opportunistic infection (i.e. TB). The caregivers' social demographic characteristics, age, sex, level of education, income, and marital status will be the independent variables.

Data processing and analysis

We will enter data into Excel™ software and analyze it using Stata™ software. Descriptive statistics will summarize continuous data, such as the mean, median, standard deviation, and interquartile range. We will use frequencies and percentages to summarize categorical data. Multiple statistical tests and methods will be employed. The univariate analysis will typically utilize Pearson's chi-squared correlation or Fisher's exact test, where appropriate. As for the primary outcome (mean difference in the viral load of HIV-positive children with VLS), we will use an independent *t*-test to compare the means of participants in the intervention and control arms. We will develop a multivariable regression model, adjusting for clustering and other confounders for potential confounding. We will use proportions and tests for different proportions as measures of improvement using the difference in difference. All statistical tests will be two-sided and $P < 0.05$ will be considered statistically significant.

Dissemination of results

We will present the findings from this study to the Simiyu Regional Health Management Team. We will also present this study at the President's Office, Regional Administrative Secretary, and Local Government's (PO-RALG) Directorate of Health, Nutrition, and Social Welfare. We will present the

Downloaded from https://academic.oup.com/biomethods/article/7/1/bpac002/6500270 by guest on 23 August 2023

finding at local and international conferences and publish the study in an international peer-reviewed journal.

Ethical clearance and consent to participate

In the first stage, we will obtain the ethical clearance and registration for this study from the Dodoma University Ethical Board. In the second stage, we will require permission to use the CTC2 database from the Ministry of Health. We will seek consent from other authorities in the third phase including the PO-RALG. In the fourth stage, we will seek permission from the person in-charge of health facilities. In the last step, we will seek approval and written consent from participants. Our study was registered in the Pan African Clinical Trial Registry with a registration number PACTR202111867711522.

Discussion

This Konga model study will be the first cluster-randomized clinical control trial study in Tanzania using the community as a basis for addressing challenges in VLS. The existing community of PLHIV will enhance ART adherence with VLS in children.

A systematic review of community-based interventions highlighted the importance of carefully designing organization-based HIV prevention interventions in a way that would improve their effectiveness and efficiency [33]. The current study will use community participation to promote retention and adherence to treatment, focusing on home-based follow-up and psychosocial and peer support [29]. Several studies have demonstrated that poor adherence and retention in ART care detract from VLS among children [34–37] living with HIV. In this intervention, we will use the Kong to promote retention and adherence to ART among children receiving ART to reduce their viral loads.

Opportunistic infections, such as TB, have been shown to hamper VLS in children receiving ART [14, 19, 20, 38]. Another study demonstrated that the initiation of ART in children reduces the incidence of TB [24]. The Konga model will include active home visits to screen children for TB and other comorbidities in the control arm. The screening will help to identify early infection [24] and facilitate referral to the treatment point.

HIV-infected children potentially suffer from mental-health disorders that result in poor quality of life, HIV disease progression; poor compliance and increased mortality [39]. Studies have demonstrated that psychiatric morbidity in HIV-infected children is higher than that in children in the general population. These studies recommended that there is a need to incorporate psychiatric liaison service routine care for HIV-infected children [40–42]. The personnel from the Konga will visit the children and their families to provide psychosocial counseling and support.

Data availability

Not applicable because this is a protocol manuscript which contain no any data.

Acknowledgement

The authors would like to acknowledge the Regional Health Management of the Simiyu region headed by Dr. Boniphase Marwa for their contribution to this project.

Author contribution

M.K., P.P., and L.K.K.: study conception and design. M.K.: draft protocol preparations. P.P., L.K.K., and N.A.K. reviewed the protocol, while L.K.K. and P.P. approved the final version of the protocol.

Funding

This research protocol has not received any specific funding from any organization.

Conflict of interest statement. None declared.

References

1. World Health Organization. *HIV/AIDS Fact Sheet* [Online], 2021. Available at https://www.who.int/news-room/fact-sheets/detail/hiv-aids (30 November 2021 date last accessed).
2. Alimonti JB, Ball TB, Fowke KR. Mechanisms of CD^{4+} T lymphocyte cell death in human immunodeficiency virus infection and AIDS. *J Gen Virol*. 2003;**84**:1649–61. https://doi.org/10.1099/vir.0.19110-0
3. World Health Organization. *Consolidated Guidelines on the Use of Antiretroviral Drugs for Treating and Preventing HIV Infection Recommendations for a Public Health Approach*. 2nd edn, 2016. Available at https://www.who.int/hiv/pub/arv/arv-2016/en/ (21 September 2019, date last accessed).
4. Tanzania Commission for AIDS (TACAIDS) and Zanzibar AIDS Commission (ZAC). *Tanzania HIV Impact Survey (THIS) 2016–2017: Final Report* [Online]. Tanzania: Dar es Salaam. Available at https://www.nbs.go.tz/index.php/en/census-surveys/health-statistics/hiv-and-malaria-survey/382-the-tanzania-hiv-impact-survey-2016-2017-this-final-report (21 September 2019, date last accessed).
5. Gona PN, Gona CM, Ballout S *et al.* Burden and changes in HIV/AIDS morbidity and mortality in Southern Africa Development Community Countries, 1990–2017. *BMC Public Health* 2020;**20**:867. https://doi.org/10.1186/s12889-020-08988-9
6. Boyd AT, Oboho I, Paulin H *et al.* Addressing advanced HIV disease and mortality in global HIV programming. *AIDS Res Ther* 2020;**17**:40. https://doi.org/10.1186/s12981-020-00296-x
7. Girum T, Yasin F, Wasie A *et al.* The effect of the "universal test and treat" program on HIV treatment outcomes and patient survival among a cohort of adults taking antiretroviral treatment (ART) in low-income settings of the Gurage zone, South Ethiopia. *AIDS Res Ther* 2020;**17**:19. https://doi.org/10.1186/s12981-020-00274-3
8. World Health Organization. *Global Health Sector Response to HIV, (2000–2015). Focus on Innovations in Africa: Progress Report*. Geneva: World Health Organization, 2015. Available at https://apps.who.int/iris/handle/10665/198065 (17 October 2021, date last accessed).
9. ——. *HIV/AIDS Fact Sheet*, 2020. Available at https://www.who.int/health-topics/hiv-aids (April 2021, date last accessed).
10. Ali JH, Yirtaw TG. Time to viral load suppression and its associated factors in a cohort of patients taking antiretroviral treatment in East Shewa zone, Oromiya, Ethiopia, 2018. *BMC Infect Dis* 2019;**19**:1084. https://doi.org/10.1186/s12879-019-4702-z
11. Abrams EJ, Woldesenbet S, Soares SJ *et al.* Despite access to antiretrovirals for prevention and treatment, high mortality rates persist among HIV-infected infants and young children.

Downloaded from https://academic.oup.com/biomethods/article/7/1/bpac002/6500270 by guest on 23 August 2023

Pediatr Infect Dis J 2017;**36**:595–601. https://doi.org/10.1097/INF.0000000000001507
12. Anigilaje EA, Aderibigbe SA. Mortality in a cohort of HIV-infected children: A 12-month outcome of antiretroviral therapy in Makurdi, Nigeria. *Adv Med* 2018;**2018**:1. https://doi.org/10.1155/2018/6409134
13. Kakkar FB, Lee T, Hawkes MTD *et al*. Challenges to achieving and maintaining viral suppression among children living with HIV. *AIDS* 2020;**34**:687–97. https://doi.org/10.1097/QAD.0000000000002454
14. Bulage L, Ssewanyana I, Nankabirwa V *et al*. Factors associated with virological non-suppression among HIV-positive patients on antiretroviral therapy in Uganda, August 2014–July 2015. *BMC Infect Dis* 2017;**17**:326. https://doi.org/10.1186/s12879-017-2428-3
15. Jiamsakul A, Kariminia A, Althoff KN *et al*. HIV viral load suppression in adults and children receiving antiretroviral therapy—results from the IDEA Collaboration. *J Acquir Immune Defic Syndr* 2017;**76**:319–29. https://doi.org/10.1097/QAI.0000000000001499
16. Teasdale CA, Sogaula N, Yuengling KA *et al*. HIV viral suppression and longevity among a cohort of children initiating antiretroviral therapy in Eastern Cape, South Africa. *J Intern Aids Soc* 2018;**21**:e25168.
17. President's Emergency Plan for AIDS Relief. *Tanzania Country Operational Plan COP2017 Strategic Direction Summary*, 2 March, 2017. Available at https://tz.usembassy.gov/wp-content/uploads/sites/258/2017/07/TZ-COP-2017-SDS-FINAL-Update-29June2017.pdf (3 November 2020, date last accessed).
18. ——. *Tanzania Country Operational Plan COP2019 Strategic Direction Summary*, May 10, 2019. Available at https://www.state.gov/wp-content/uploads/2019/09/Tanzania_COP19-Strategic-directional-Summary_public.pdf (5 November 2020, date last accessed).
19. Barnabas RV, Webb EL, Weiss HA *et al*. The role of co-infections in HIV epidemic trajectory and positive prevention: A systematic review and meta-analysis. *AIDS* 2011;**25**:1559–73. https://doi.org/10.1097/Q.A.D.0b013e3283491e3e
20. Martelli G, Antonucci R, Mukurasi A *et al*. Adherence to antiretroviral treatment among children and adolescents in Tanzania: Comparison between pill count and viral load outcomes in a rural context of Mwanza region. *PLoS ONE* 2019;**14**:e0214014.https://doi.org/10.1371/journal.pone.0214014
21. Bartelink IH, Savic RM, Dorsey G *et al*. The effect of malnutrition on the pharmacokinetics and virologic outcomes of lopinavir, efavirenz and nevirapine in food insecure HIV-infected children in Tororo, Uganda. *Pediatr Infect Dis J* 2015;**34**:e63–e70. https://doi.org/10.1097/INF.0000000000000603
22. Muenchhoff M, Healy M, Singh R *et al*. Malnutrition in HIV-infected children is an indicator of severe disease with an impaired response to antiretroviral therapy. *AIDS Res Hum Retroviruses* 2018;**34**:46–55. https://doi.org/10.1089/AID.2016.0261
23. Gupta RK, Lucas SB, Fielding KL *et al*. Prevalence of tuberculosis in post-mortem studies of HIV-infected adults and children in resource-limited settings: A systematic review and meta-analysis. *AIDS* 2015;**29**:1987–2002. https://doi.org/10.1097/QAD.0000000000000802
24. Anígilájé EA, Aderibigbe SA, Adeoti AO *et al*. Tuberculosis, before and after antiretroviral therapy among HIV-infected children in Nigeria: What are the risk factors? *PLoS ONE* 2016;**11**:e0156177.
25. Geoffrey F, Shaik N, Brian E *et al*. Improved virological suppression in children on antiretroviral treatment receiving community-based adherence support: A multicentre cohort study from South Africa. *AIDS Care* 2014;**26**:448–53. https://doi.org/10.1080/09540121.2013.855699
26. Munthali T, Hachizovu S, Washington M. The last stride to 90–90–90: Improving viral suppression in children (under 16 years) through community-based ART in Zambia. *Health Press Zambia Bull* 2020;**4**:6–9.
27. Kigen HT, Galgalo T, Githuku J *et al*. Predictors of loss to follow up among HIV-exposed children within the prevention of mother to child transmission Cascade, Kericho County, Kenya, 2016. *Pan Afr Med J* 2018;**30**:178. https://doi.org/10.11604/pamj.2018.30.178.15837
28. Modjarrad K, Vermund SH. Effect of treating co-infections on HIV-1 viral load: A systematic review. *Lancet Infect Dis* 2010;**10**:455–63. https://doi.org/10.1016/S1473-3099(10)70093-1
29. Mukherjee JS, Barry D, Weatherford RD *et al*. Community-based ART programs: Sustaining adherence and follow-up. *Curr HIV/AIDS Rep* 2016;**13**:359–66. https://doi.org/10.1007/s11904-016-0335-7
30. Wouters E, Van Damme W, van Rensburg D *et al*. Impact of community-based support services on antiretroviral treatment programme delivery and outcomes in resource-limited countries: A synthetic review. *BMC Health Serv Res* 2012;**12**:194. https://doi.org/10.1186/1472-6963-12-194
31. United Nations Program on HIV/AIDS. *Promising Practices in Community Engagement to Eliminate New HIV Infections among Children by 2015 and Keeping Their Mothers Alive*. UNAIDS Case Study/2012, 2015. Available at https://www.unaids.org/sites/default/files/media_asset/20120628_JC2281_PromisingPractices CommunityEngagements_en_0.pdf (2 March 2021, date last accessed).
32. National AIDS Control Program. *National Guideline for the Management of HIV and AIDS*. 6th edn., 2017. Available at http://www.nacp.go.tz/download/national-guidelines-for-the-management-of-hiv-and-aids/ (4 October 2018, date last accessed).
33. Ibrahim S, Sidani S. Community-based HIV prevention intervention in developing countries: A systematic review. *Adv Nurs* 2014;**17496**.
34. Abreu JC, Vaz SN, Netto EM *et al*. Virological suppression in children and adolescents is not influenced by genotyping, but depends on optimal adherence to antiretroviral therapy. *Braz J Infect Dis* 2017;**21**:219–25. https://doi.org/10.1016/j.bjid.2017.02.001
35. Gaitho D, Kemunto D, Kinoti F. Determinants of viral non-suppression among children in an HIV program in Kenya: A cross-sectional study. *Sex Transm Infect* 2019;**95**:A128–A129.
36. Yihun BA, Kibret GD, Leshargie CT. Incidence and predictors of treatment failure among children on first-line antiretroviral therapy in Amhara Region Referral Hospitals, northwest Ethiopia 2018: A retrospective study. *PLoS ONE* 2019;**14**:e0215300.https://doi.org/10.1371/journal.pone.0215300
37. Zoufaly A, Fillekes Q, Hammerl R *et al*. Prevalence and determinants of virological failure in HIV-infected children on antiretroviral therapy in rural Cameroon: A cross-sectional study. *Antivir Ther* 2013;**18**:681–90. https://doi.org/10.3851/IMP2562
38. Afrane AKA, Goka BQ, Renner L *et al*. HIV virological non-suppression and its associated factors in children on antiretroviral therapy at a major treatment centre in Southern Ghana: A cross-sectional study. *BMC Infect Dis* 2021;**21**:731. https://doi.org/10.1186/s12879-021-06459-z
39. Scharko AM. DSM psychiatric disorders in the context of pediatric HIV/AIDS. *AIDS Care* 2006;**18**:441–5. https://doi.org/10.1080/09540120500213487

Downloaded from https://academic.oup.com/biomethods/article/7/1/bpac002/6500270 by guest on 23 August 2023

40. Kehinde OB, Muideen OB, Bassey EE *et al*. Psychological complications associated with HIV/AIDS infection among children in South–South Nigeria, sub-Saharan Africa. *Cogent Med* 2017;**4**: 1372869.https://doi.org/10.1080/2331205X.2017.1372869
41. Kamau JW, Kuria W, Mathai M *et al*. Psychiatric morbidity among HIV-infected children and adolescents in a resource-poor Kenyan urban community. *AIDS Care* 2012;**24**:836–42. https://doi.org/10.1080/09540121.2011.644234
42. Mellins CA, Elkington KS, Leu CS *et al*. Prevalence and change in psychiatric disorders among perinatally HIV-infected and HIV-exposed youth. *AIDS Care* 2012;**24**:953–62. https://doi.org/10.1080/09540121.2012.668174

Downloaded from https://academic.oup.com/biomethods/article/7/1/bpac002/6500270 by guest on 23 August 2023
